# The neurophysiology of continuous action monitoring

**DOI:** 10.1016/j.isci.2023.106939

**Published:** 2023-05-22

**Authors:** Saskia Wilken, Adriana Böttcher, Nico Adelhöfer, Markus Raab, Sven Hoffmann, Christian Beste

**Affiliations:** 1 General Psychology: Judgment, Decision Making, and Action, Institute of Psychology, University of Hagen, Hagen, Germany; 2 Cognitive Neurophysiology, Department of Child and Adolescent Psychiatry, Faculty of Medicine, TU Dresden, Dresden, Germany; 3 University Neuropsychology Center, Faculty of Medicine, TU Dresden, Dresden, Germany; 4 Donders Institute of Cognition and Behaviour, Radboud University Medical Center, Nijmegen, the Netherlands; 5 Performance Psychology, Institute of Psychology, German Sport University Cologne, Cologne, Germany; 6 School of Applied Sciences, London South Bank University, London, UK

**Keywords:** Sensory neuroscience, Cognitive neuroscience

## Abstract

Monitoring actions is essential for goal-directed behavior. However, as opposed to short-lasting, and regularly reinstating monitoring functions, the neural processes underlying continuous action monitoring are poorly understood. We investigate this using a pursuit-tracking paradigm. We show that beta band activity likely maintains the sensorimotor program, while theta and alpha bands probably support attentional sampling and information gating, respectively. Alpha and beta band activity are most relevant during the initial tracking period, when sensorimotor calibrations are most intense. Theta band shifts from parietal to frontal cortices throughout tracking, likely reflecting a shift in the functional relevance from attentional sampling to action monitoring. This study shows that resource allocation mechanisms in prefrontal areas and stimulus-response mapping processes in the parietal cortex are crucial for adapting sensorimotor processes. It fills a knowledge gap in understanding the neural processes underlying action monitoring and suggests new directions for examining sensorimotor integration in more naturalistic experiments.

## Highlights

• Continuous action monitoring is crucial for everyday goal-directed behavior
• We investigate the underlying neurophysiology with a new EEG paradigm
• Modulation of alpha, beta, and theta band activity in widespread cortical regions

## Graphical abstract

**Figure:**
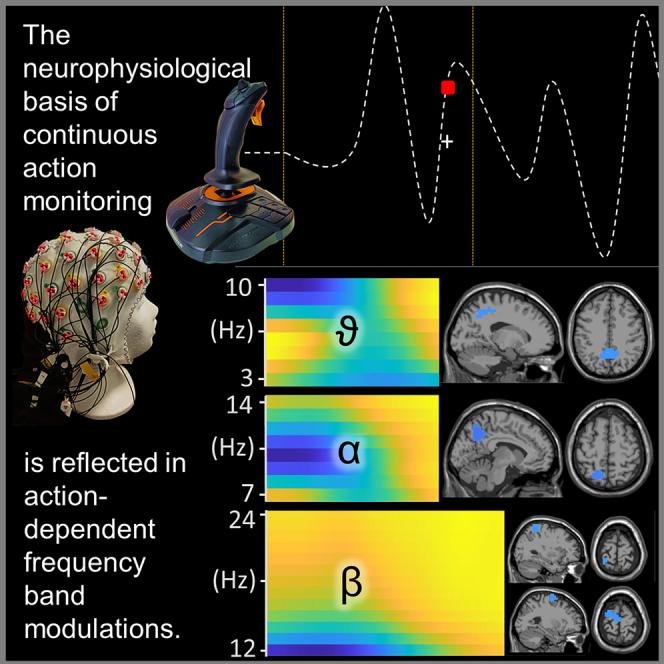

## Introduction

The monitoring of one’s actions is a central requirement for goal-directed behavior. Despite its ubiquitous nature, the underlying neural mechanisms are only incompletely understood. Existing conceptions on action monitoring, and especially considerations on the functional relevance of neurophysiological correlates of action monitoring, are based on analyses/experiments during relatively short-lasting monitoring or demands that reinstate regularly.^1^^,^^2^ However, in the rarest cases, real-time action monitoring is short-lasting and regularly reinstates in discretized occasions.^1^ Instead, action monitoring is performed continuously over several seconds or even longer with little prior knowledge about the intensity with which action monitoring processes are demanded, such as in driving.^3^^,^^4^^,^^5^ Moreover, actions do not consist solely of sequential action outcome evaluation. Instead, they require online adjustment of movements and continuous prediction of motor program outcomes (e.g., playing soccer and controlling the ball while passing opponents). Thus, it is tentative whether knowledge from classical experiments and the involved neurophysiological functions^6^ can be generalized to other more naturalistic settings. There is thus an urgent need to clarify the neural mechanisms of continuous action monitoring, which is done in the current study through pursuit-tracking tasks and an in-depth analysis of neurophysiological processes applying various electroencephalography (EEG) analysis methods. We combine the analysis of oscillatory dynamics, as a fundamental communication mechanism of the human brain,^7^ with high-resolution time-domain event-related potential (ERP) analysis to achieve a detailed overview of the neurophysiological correlates of continuous action monitoring.

Previous studies examining action monitoring during reinstating and short-lasting demands have consistently found that theta band activity plays a role,^8^^,^^9^^,^^10^ such that it likely serves as a “surprise signal” necessary to adapt actions.^8^ The theta-related surprise signal is used to alter learning processes to adjust behavioral strategy and the need to increase cognitive control.^8^ The importance of medial frontal theta band activity during action monitoring relates to biophysical principles of high-amplitude/low-frequency oscillations.^11^ These types of oscillations are optimal for synchronizing activities between multiple remote functional neuroanatomical regions.^8^ Of note, theta band activity and other EEG correlates associated with the medial frontal and superior parietal cortex are central for integrating sensory and motor processes,^12^^,^^13^^,^^14^ which well-reflects overarching conceptualizations of the medial frontal cortex.^15^ However, theta band activity also plays a role during attentional selection processes^16^^,^^17^ and especially so in parietal cortices, which are known to be involved in binding sensory and motor information into a sensorimotor representation (program) necessary for goal-directed action.^18^ Accordingly, these brain structures should also reveal theta band activity modulation during continuous action monitoring. Crucially, when considering the role of theta band activity, beta band activity needs to be considered as well since both are jointly involved in motor control.^19^ Moreover, beta band activity has been associated with top-down controlled processing,^20^ including attention to upcoming motor tasks.^21^^,^^22^ It may thus reflect premotor mechanisms guiding motor actions.^23^ Therefore, beta band activity may reflect the computation of the “status quo,”^24^ meaning that beta band activity reflects the continuous maintenance of a sensorimotor program depending on the prediction of what is likely to happen soon, to adapt the sensorimotor program.^25^^,^^26^ All these aspects make it likely that beta band activity plays an essential role during the continuous monitoring of actions. However, a conceptually related role has been ascribed to alpha band activity.^27^ Alpha band activity likely reflects the gating and controlled access of incoming information to a domain-general “knowledge system.”^27^ Supposedly, alpha band activity plays a role in anticipatory attentional processes needed to maintain target information.^27^ Based on these considerations, we examine the theta, alpha, and beta band activity dynamics during continuous action monitoring combining time-frequency and EEG-beamforming methods in a pursuit-tracking task in the current study. We hypothesize that these activities are modulated during continuous action monitoring and that frontoparietal cortices reflect these dynamics. The latter is likely because the intraparietal sulcus and the dorsal premotor cortex probably constitute the major cortical network to control complex movement kinematics.^26^

Despite oscillatory activity reflecting a fundamental information processing principle in the human brain,^7^ the limited temporal resolution of the time-frequency analytical approach is a drawback. Performance in pursuit tracking varies considerably^28^ depending on acquired implicit knowledge and the predictability of the target trajectory.^3^ Especially after trajectory direction changes, adaptive processes have to be triggered to reduce spatial tracking errors. Thus, there are discrete points where adaptive processes are required. The analysis of time-domain EEG data is necessary to capture the neurophysiological substrates of these temporal dynamics. An ERP component capturing these processes may be the error-related negativity (ERN)^29^^,^^30^^,^^31^^,^^32^^,^^33^ originating from medial frontal cortices.^34^ The ERN reflects a comparison process between the desired and the achieved action outcome,^35^ which is also required during continuous sensorimotor processing.^26^ It is commonly accepted that such aspects of performance monitoring are necessary when adjusting action-to-action outcomes.^34^ In addition, it is also mandatory to increase processing capacities to adapt complex movements when sensorimotor processes have to be adapted (because they deviate too much from the desired state). Such resource allocation processes are likely reflected by the P2 ERP component,^36^^,^^37^^,^^38^ while adaptations in the mapping of the stimulus on the appropriate response are reflected by the P3 ERP component.^39^^,^^40^ Since monitoring and adaptation are highly dynamic,^41^^,^^42^ we also relate tracking and EEG dynamics^43^ by relating single-trial EEG dynamics of the ERP parameters mentioned previously and tracking performance.

To experimentally disentangle demands on continuous action monitoring processes and to examine their underlying neurophysiology in detail, participants performed two consecutive tasks employing a pursuit-tracking paradigm requiring continuous tracking of a moving target with a cursor using a joystick: In the first task, we examined the execution of complex movements requiring online sensorimotor adaptation. For that, one trajectory segment was kept constant across trials, and two randomly generated trajectory segments were appended at the end and beginning of this constant trajectory, respectively. Comparisons were performed between the constant and random trajectory segments. The second task tested the prediction of visual trajectories in preparation for actions using segments without visual feedback about the cursor location. Like in the first task, the target trajectory was a concatenation of two random parts and constant part in the middle. At regular intervals, the cursor was occluded. These paradigms are standard in the field of pursuit tracking.^3^^,^^4^^,^^5^ In this study, we investigate the association of alpha, beta, and theta band activity with the execution of complex movements with different sensorimotor demands, i.e. predictability of the target trajectory and visibility of the cursor. Furthermore, we investigate ERPs related to target direction changes with different sensorimotor demands. Finally, we will examine the predictability of the ERP amplitudes by the behavioral tracking performance.

## Results

**Figure 1:**
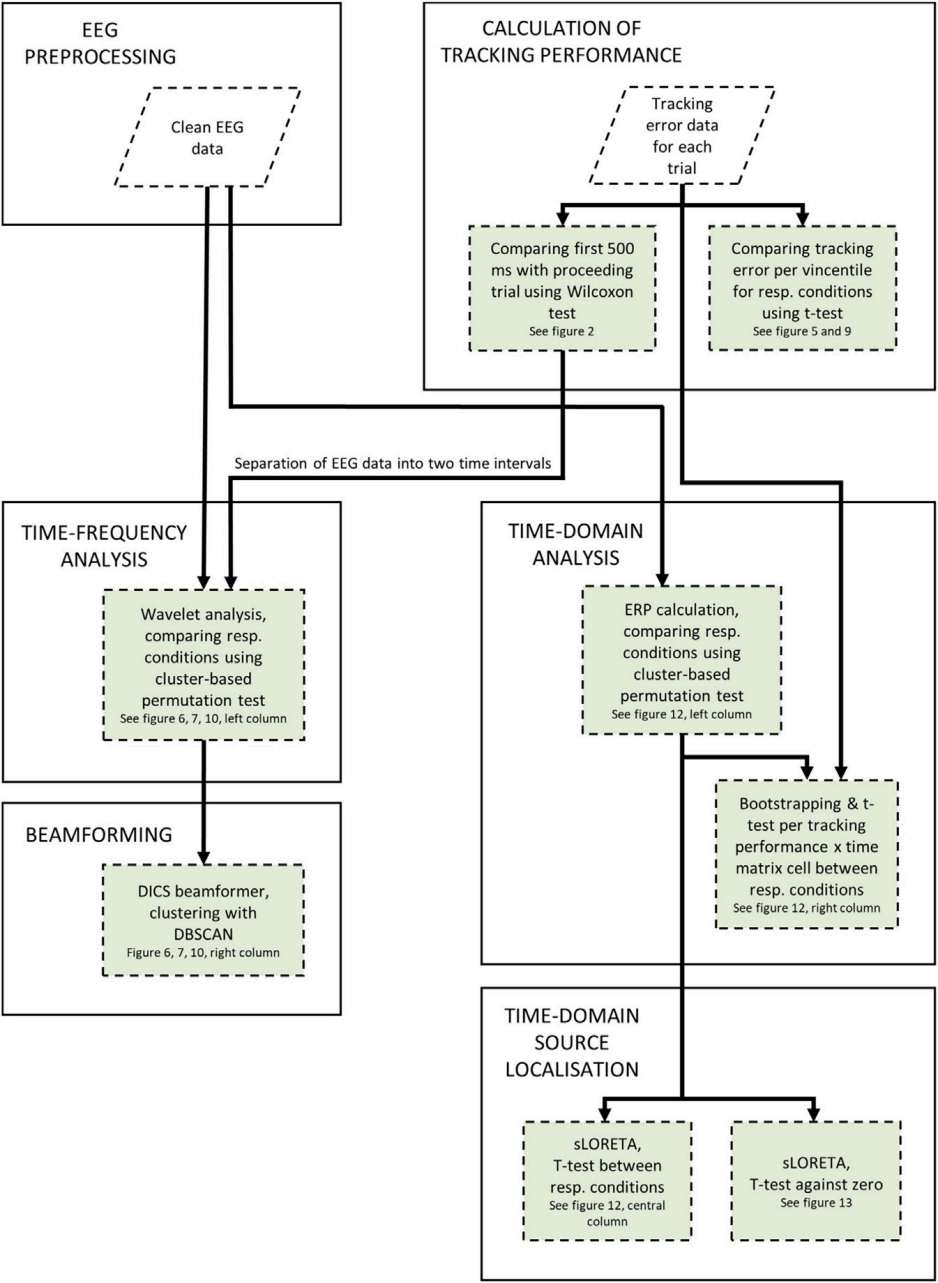
Flow diagram overview of analyses After general preprocessing of the EEG data, we split EEG analyses in two analysis strands: the time-frequency domain and the time domain analyses which both culminate respectively in a suitable source localization. The analysis of the behavioral data was used in conjunction with the time-domain EEG data. Additionally, exploring the observed differences in tracking behavior during the first 500 ms of a trial as compared to the proceeding trial led to the introduction of an additional contrast in the time-frequency analyses. Analyses are shown in green shaded rectangles, data are depicted in white diamonds.

To gain an overview of the order of data analysis steps for the current study, please refer to Figure 1. The analyses were performed for both tasks. After summarizing explorative findings in the behavioral data that shaped our analysis of task 1, we present the results grouped by the two tasks.

### General behavioral data

**Figure 2:**
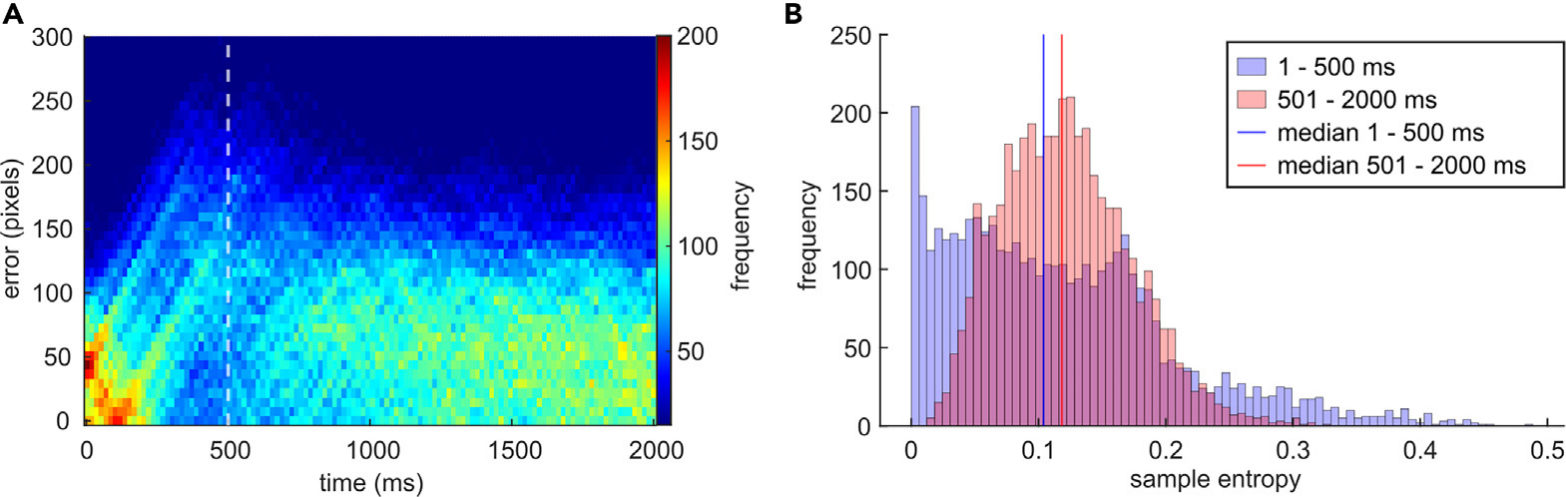
Visualization of change in tracking error between first 500 ms and following 1500 ms Figure (A) shows the distribution of all subjects' tracking error magnitudes (y axis) over time (x axis) during the first 2000 ms of all trials, with the color scale denoting the frequency (number of trials) of tracking error magnitudes per frame. A more peaked and more predictable frequency distribution can be observed during the first 500 ms of the trial compared to the rest. Figure (B) depicts the quantification of this observation using sample entropy as a measure of time-series predictability. It shows the frequency (y axis) of sample entropy values (x axis) of all subjects and all trials for the first 500 ms (blue) compared to the following 1500 ms (red). The median of the sample entropy values in the first 500 ms interval (blue line) is significantly smaller than the median of the 501–2000 ms interval sample entropy values (red line).

In both tasks, the participants were asked to continuously track a steadily moving target with a cursor along a trajectory using a joystick. First, we investigated whether the tracking data revealed changes in tracking behavior patterns across the trial. We calculated the tracking error for each frame by subtracting the y-position of the cursor on the screen from the y-position of the target on the screen. Then, we inspected the distribution of the absolute tracking error for each frame over one trial, across all trials of all participants. As shown in Figure 2A, the pattern of tracking errors differed in the first 500 ms interval of the trial compared to the later trial interval.

**Figure 3:**
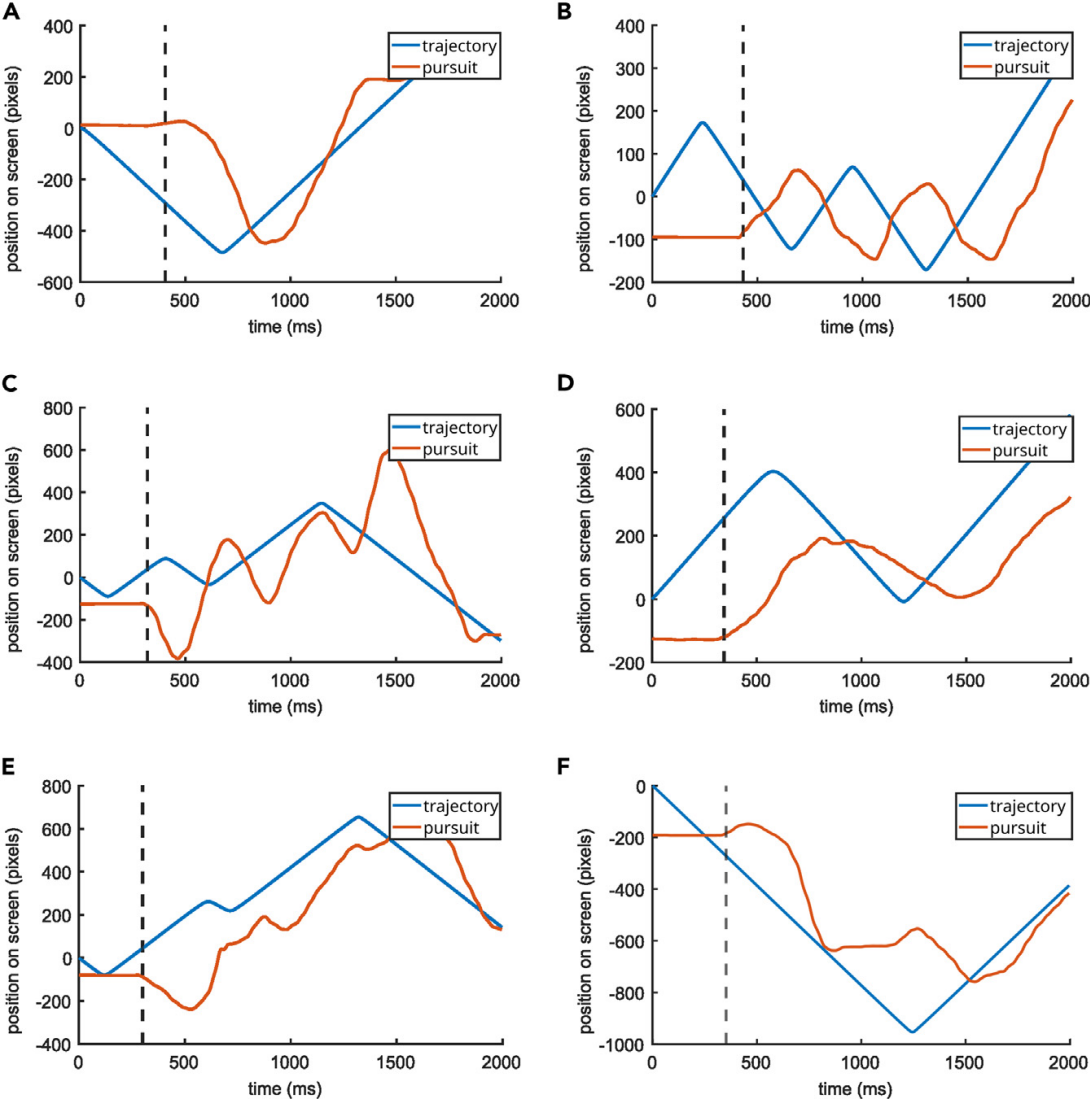
Example trials illustrating the difference in tracking behavior during the first 500 ms vs. the following 1500 ms This figure shows randomly picked trials (6, figure parts A–F) from various subjects. As can be seen, subjects do not immediately move the joystick in pursuit of the target after the target started moving, which is why the tracking error during this time is mainly due to the course of the random trajectory. The dashed line indicates the moment the participant started moving the joystick in pursuit of the target. Note that the apparently linear sections of the trajectory are due to omitting x-coordinate information in this plot so that time can be plotted with equidistant tick marks on the x axis. The trajectory on screen had no linear sections.

To quantify this difference, we calculated the sample entropy^44^ of the z-transformed tracking error of the first 500 ms interval of each trial of each participant respectively and the z-transformed tracking error of the following 1500 ms interval of each trial (see Figure 2B). Sample entropy quantifies the predictability of a time series without requiring assumptions about its distribution. We used a Wilcoxon rank-sum test to compare sample entropy values (one for each subject) from the first 500 ms interval to the sample entropy values of the following 1500 ms interval. We found that entropy is significantly lower in the first (median = 0.104) compared to the second (median = 0.122) interval (Z = −3.65, p < 0.001, r = −0.67). Upon visually inspecting tracking and pursuit of the individual trials, we noticed that participants frequently did not actively move the cursor from the middle line during the first 500 ms interval of a trial (Figure 3). At the start of the trial, the difference between the cursor position and target position was more determined by the target path than by the rest of the trial. Nevertheless, the discrepancy was evident for the participants due to the visual input. Due to the observed differences between the first 500 ms interval and the remaining part of the trial, the analysis of the neurophysiological (EEG) data was performed separately for the first 500 ms and the remaining time of the trial.

#### Task 1: Online sensorimotor adaptation

**Figure 4:**
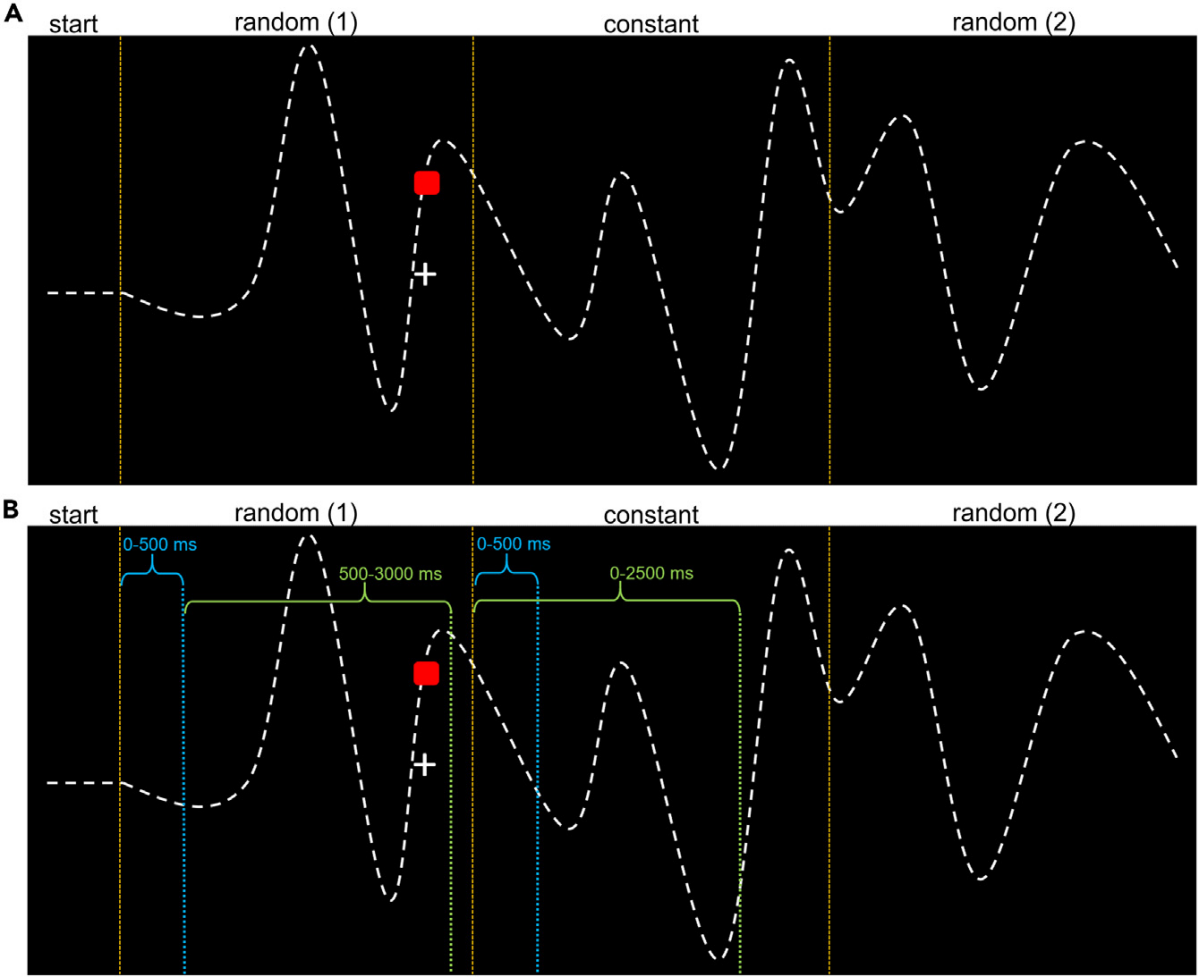
One trial of our pursuit-tracking paradigm measuring online sensorimotor adaptation and the segmentation for EEG data analysis (A) Participants were asked to follow a continuously moving target (a red square) along an invisible trajectory, depicted as a white dashed line, using a cursor (a white cross). Horizontal cursor movements were locked to the target. Following a start vector at the beginning of each trial, the target trajectory consisted of three segments connected via cubic splines. The first and last segments were randomly generated for each trial, whereas the middle segment was repeated in every trial. (B) Since we found significant differences in the tracking behavior pattern during the first 500 ms of each trial compared to the rest of the trial, we segmented the EEG into two intervals for the random and constant trajectory, respectively. The first interval consisted of the first 500 ms of the random and constant trajectory segment (depicted in blue). The second interval compared 500–3000 ms for the random trajectory segment to 0–2500 ms for the constant segment (depicted in green).

In the first task, participants tracked the target with a cursor along a trajectory concatenated from two random trajectory segments (at the trial start and end) and one constant, i.e., repeated segment in between (see trial overview in Figure 4A).

### Behavioral data

**Figure 5:**
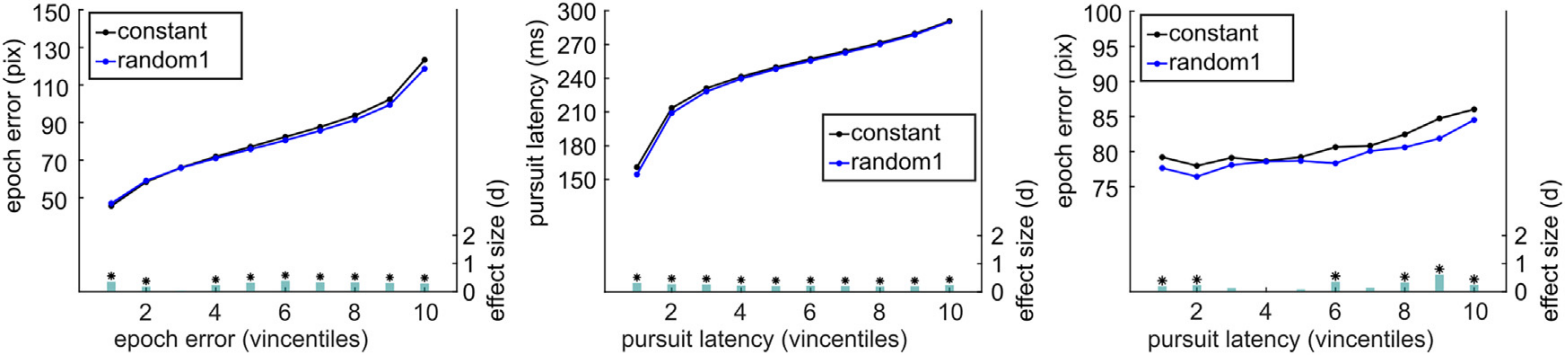
Vincentiles of tracking performance measures by condition constant vs. random The two left plots visualize which magnitudes the error measures assume (y axis), per vincentile (dots) and separated by condition (blue resp. black line). Additionally, at the bottom, there is a bar plot indicating whether there are significant differences between conditions and how large that difference is. The smallest, still significant t-value is *t*(29) = -1.79, p = 0.03. Note that the effect size values are absolute values (not considering direction of effect) for easier visualization. The rightmost plot shows the epoch error in pixels sorted by the vincentiles of pursuit latency per condition. It visualizes a slightly positive association between the two variables in task 1 for constant as well as random trajectory segments.

Two measures were used to quantify the accuracy of pursuit tracking: First, the mean of the vertical difference between cursor and target was calculated, thus measuring the tracking error (accuracy) for the respective epoch (epoch error). Second, for each target direction change, the latency of the following matching pursuit direction change was calculated (pursuit latency). This reflects the time needed to adjust the cursor movement to a change in the target trajectory. First, we were curious to know how differences between conditions change with increasing magnitude of tracking error and pursuit latency. To this end, we ordered the tracking error and pursuit latency into ten vincentiles per subject. For data analysis, we took 5,000 bootstrap samples from each vincentile and performed paired t-tests on the respective vincentiles, comparing the error measure magnitudes between the first random and the constant trajectory segments. Both, tracking error and pursuit latency were significantly larger in the constant condition in almost all vincentiles (except for the first three epoch error vincentiles) (see Figure 5), indicating slower reaction times to changes in the target direction and lower tracking accuracy in the constant segments compared to the random segments. The effect sizes remain relatively stable across vincentiles, ranging from d = −0.36 to d = 0.30 for epoch error and from d = 0.19 to d = 0.31 for pursuit latency. Finally, we sorted the epoch error by the vincentiles of pursuit latency to obtain a measure of association between the two tracking performance measures. We found a slightly positive association between the epoch error and pursuit latency as can be seen in Figure 5. This means that lower accuracy is associated with slower reaction times to target direction changes. The size of this effect ranges from d = 0.07 to d = 0.61.

### Time-frequency decomposition and EEG beamforming analyses

**Figure 6:**
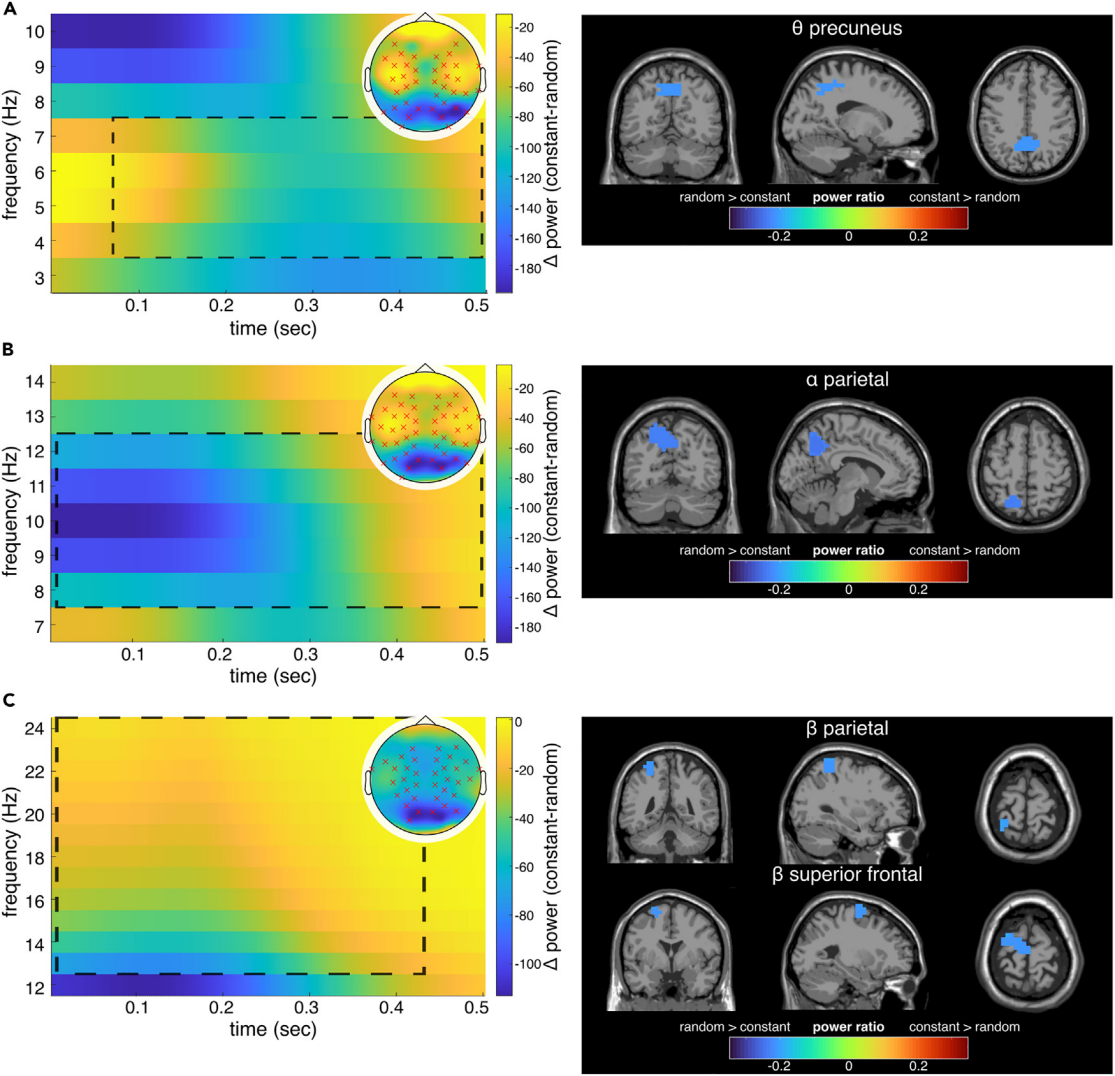
Results of the time-frequency and beamforming analysis for the contrast of constant and random trajectory segment (constant-random) in the first interval of the pursuit-tracking paradigm Frequency power differences for significant electrodes in cluster-based permutation test on the left. The topography for the respective power difference is shown in the top right corner. Significant electrodes are highlighted with red crosses for negative and black crosses for positive differences. Significant negative differences (random > constant) were found for the alpha, beta, and theta band. The dashed lines approximately enclose the significant time interval and frequency range. On the right, source activity differences for the respective frequency band are depicted in the clusters identified by the DBSCAN algorithm. (A) Theta (4–7 Hz) power differences, including the results of the beamforming analysis. Significant negative power differences were found for 68–500 ms. (B) Alpha (8–12 Hz) power differences, including the results of the beamforming analysis. Significant negative differences were found for the whole time interval. (C) Beta (13–30 Hz) power differences, including the results of the beamforming analysis. Significant negative differences were found for 0–436 ms.

Based on the behavioral data analysis (cf. General behavioral data section), the EEG data recorded during the random and constant trajectory segments were analyzed separately for the first 500 ms interval and the rest of the trial. This resulted in two segments for the random trajectory: 0–500 ms as the interval for the trial start (from now on referred to as “first interval”), and 500–3000 ms for the proceeding trial (from now on named “second interval”). The EEG data for the constant trajectory segment were also split into two parts of the same lengths as the first and second intervals of the random trajectory, respectively. The first interval of the constant trajectory ranged from 0 to 500 ms, whereas the second interval ranged from 0 to 2500 ms (see Figure 2A). We chose to use the first 500 ms for both intervals twice to underline that we do not assume that there is any difference in between the EEG activity within the first 500 ms of the constant trajectory (since it is the central part of the trial) compared to the following 2500 ms (see Figure 4). Then, in preparation of time-frequency decomposition, we analyzed differences in alpha, beta, and theta band power between constant and random trajectory segments for the first (i.e., trial start) and second time interval (i.e., proceeding trial) at the sensor level using cluster-based permutation tests. This way, we identified which contrasts revealed significant differences at the sensor level. We then reconstructed the source activity for each significant contrast using dynamic imaging of coherent sources (DICS) beamforming.^45^ We calculated the normalized difference of source activity and extracted demarcated brain areas with a clustering algorithm (DBSCAN), as done in previous studies.^46^^,^^47^ The results are shown in Figure 6.

For the first 500 ms (first interval), the cluster-based permutation tests at the sensor level revealed significant differences between the constant and random trajectory in the respective first 500 ms, i.e., between the trial onset and the middle of the trial, in the alpha, beta, and theta frequency band. The test revealed one significant negative cluster in the alpha frequency band (p < 0.001) on sensor level during the whole time interval. Since the test is based on the difference between frequency power during constant and random trajectory segments (constant-random), a negative cluster indicates higher power in the random compared to the constant trajectory. On source level, DICS beamforming for constant-random revealed activity modulations in the left superior and medial parietal lobe (BA7). For the beta band, *N* = 7, significant negative clusters for constant-random were detected on the sensor level (all p < 0.001) from 0 to 436 ms. The DICS beamforming for this contrast revealed activity modulations in the left-hemispheric parietal cortex (BA5, BA7) and superior frontal areas of the left hemisphere (BA4, BA6). For the theta frequency band, the cluster-based permutation test also revealed three significant negative clusters (random > constant) at the sensor level (all p < 0.001) from 68 to 500 ms. For this contrast, the source reconstruction revealed activity modulations in the left and right precuneus, extending to superior parietal areas (BA7).

**Figure 7:**
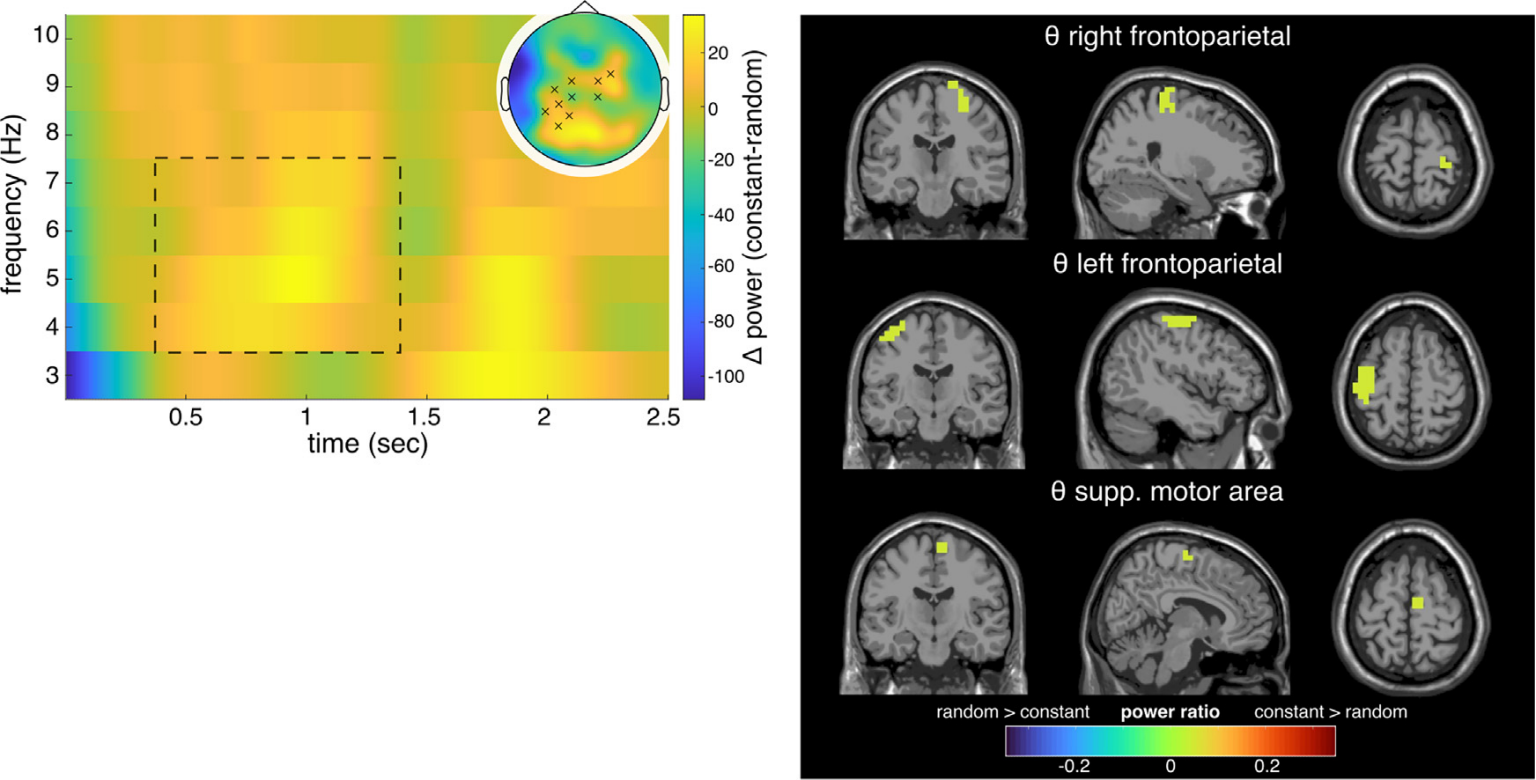
Results of the time-frequency and beamforming analysis for the contrast of constant and random trajectory (constant-random) in the second interval (i.e., proceeding trial) of the pursuit-tracking paradigm Theta band (4–7 Hz) power differences for significant electrodes in cluster-based permutation test on the left. Note that the time information on the x axis applies to the constant trajectory segment, whereas for the random trajectory segment, the time window would be 500–3000 ms (see Figure 4). The topography for the theta power difference is shown in the top right corner. Significant electrodes are highlighted with red crosses for negative and black crosses for positive differences. Significant positive power differences (constant > random) were found for 928–1944 ms. The dashed lines approximately enclose the significant time interval and frequency range. On the right, source activity differences for the theta frequency band are depicted in the clusters identified by the DBSCAN algorithm.

In the second interval, ranging from 500 to 3000 ms in the random trajectory and 0 to 2500 ms in the constant trajectory segment, we found significant differences on the sensor level only in the theta band (see Figure 7). For the theta band, two significant positive clusters (both p < 0.001) were identified on sensor level from 928 to 1944 ms for the random trajectory (or 428 to 1444 ms for the constant trajectory, due to the different time windows). Positive clusters indicate higher frequency power in the constant compared to the random segment. Reconstructing source-level activity for this contrast, activity modulations in the right frontoparietal cortex (BA4, BA5, and BA6) and the left frontoparietal cortex (BA4 and BA5) were obtained. Moreover, activity modulations were evident in the right supplementary motor area (BA4).

### Time domain analyses

In addition to testing the differences between conditions in the frequency domain, we also investigated whether there are significant differences in ERPs following target direction changes. To that end, we epoched the EEG data into 0–750 ms sections after each target direction change. Then, we performed a cluster-based permutation test over the whole epoch duration, comparing the ERPs of the random and constant condition epochs on sensor level. The test detected four significant negative clusters and eight significant positive clusters. Positive clusters indicate that the ERP in the constant trajectory segment was significantly more negative than the ERP in the random trajectory segment during the respective time interval and for the respective electrodes. Yet, none of the significant clusters could be interpreted because none surpassed our criteria for cluster selection (see STAR Methods section for details).

#### Task 2: Prediction of sensorimotor trajectories

**Figure 8:**
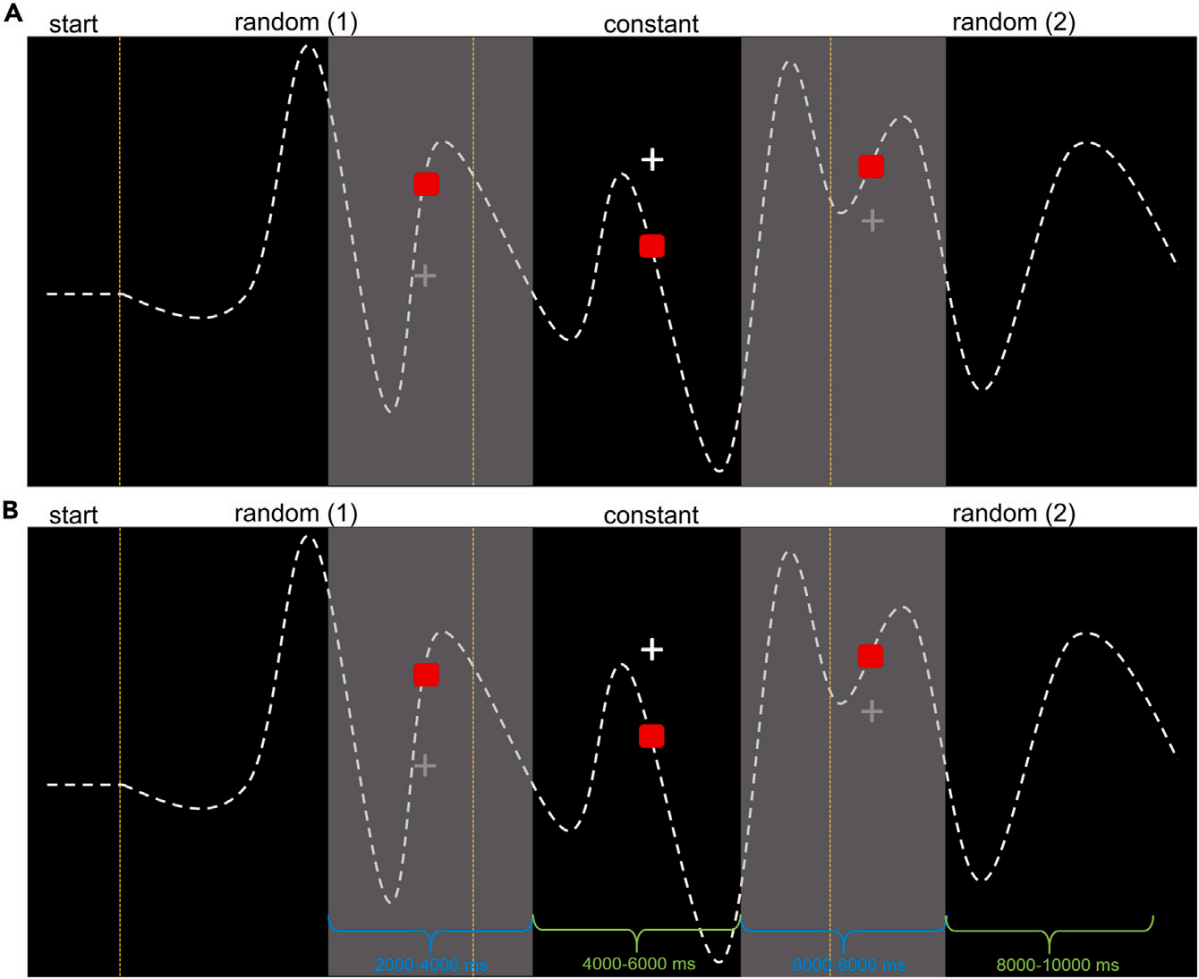
One trial of our pursuit-occlusion paradigm measuring the prediction of sensorimotor trajectories and the segmentation for EEG data analysis (A) Participants were asked to follow a steadily moving target along the concatenation of random and constant trajectory segments. The cursor was occluded for 2 s every 2 s (depicted as gray shaded areas). During the occlusion periods, participants had to predict the cursor position. (B) The EEG data were segmented according to the occlusion periods, with occluded segments depicted in blue and visible in green.

To investigate the prediction of sensorimotor trajectories, we modified our pursuit-tracking paradigm (Task 1). Again, participants were asked to follow a steadily moving target along the concatenation of trajectory segments. Importantly, the cursor moved by the participants was occluded for 2 s every 4 s (see Figure 8). During the occlusion period, participants had to predict the cursor’s current position and continue tracking the target. Therefore, we focused on these segments in the data analysis (see Figure 8).

### Behavioral data

**Figure 9:**
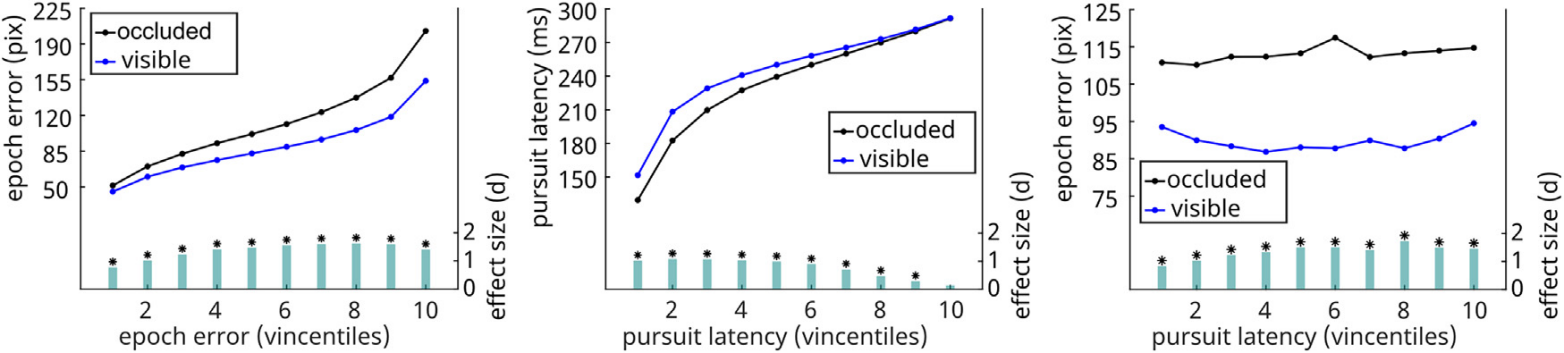
Vincentiles of tracking performance measures by condition occluded vs. visible The two left plots visualize which values the error measures assume per vincentile and separated by condition. Additionally, it is plotted whether the conditions are significantly different and how large the effect is. The smallest, still significant t-value is *t*(29) = -1.87, p = 0.02. Note that the effect size values are absolute values (not considering direction of effect) for a simpler visualization. The right plot shows the epoch error in pixels sorted into the vincentiles of pursuit latency per condition. It visualizes that there is no clear association between epoch error and pursuit latency in task two neither in the occluded nor the visible condition.

Here, we applied the same procedure as in the first task, using the epoch error and pursuit latency to quantify the accuracy of pursuit tracking. We ordered pursuit latencies and epoch errors into ten vincentiles per subject and drew 5,000 bootstrap samples per vincentile. We then performed t-tests to compare the occluded and non-occluded trajectory segments. Analogously to task one, the epoch error and pursuit latency differed significantly in almost all vincentiles (see Figure 9). For the epoch error vincentiles, effect sizes monotonously increased from the first to the eight vincentile (min *d* = 0.77, max *d* = 1.69). In each vincentile, the epoch error was larger when the cursor was occluded compared to the non-occluded condition. So, tracking accuracy is reduced when the cursor is invisible. For the pursuit latency vincentiles, we observed a monotonous reduction in effect sizes from large effects in low vincentiles, i.e., short pursuit latency, to non-significant medium effects in the highest vincentile, i.e. long pursuit latency (min *d* = −1.02, max *d* = −0.15). The pursuit latencies of every vincentile were larger in the visible condition compared to the occluded condition. In other words, seeing the cursor while tracking is associated with a slower reaction time to changes in the target movement direction. These findings likely reflect an interference of processes: tracking the cursor might interfere with the ability to respond to changes in the trajectory quickly. Finally, we also investigated how the epoch error and pursuit latency are related by sorting the epoch error by the pursuit latency vincentiles. We found that there is no clear association between epoch error and pursuit latency. However, the difference in epoch error between the conditions when sorted by pursuit latency is overall large, ranging from *d* = 0.84 to *d* = 1.72.

### Time-frequency decomposition and EEG beamforming analyses

To analyze neurophysiological processes associated with predicting sensorimotor trajectories, we analyzed differences in alpha, beta, and theta power between occluded and non-occluded trajectory segments. We reconstructed the source activity using the DICS beamformer for significant effects at the sensor level and analyzed the contrast by calculating the normalized difference between both conditions. Then, we identified source-level clusters using the DBSCAN algorithm. The cluster-based permutation test for the contrast occluded vs. visible in the theta band on the sensor level revealed four significant negative clusters for the entire time interval (all p < 0.001).

**Figure 10:**
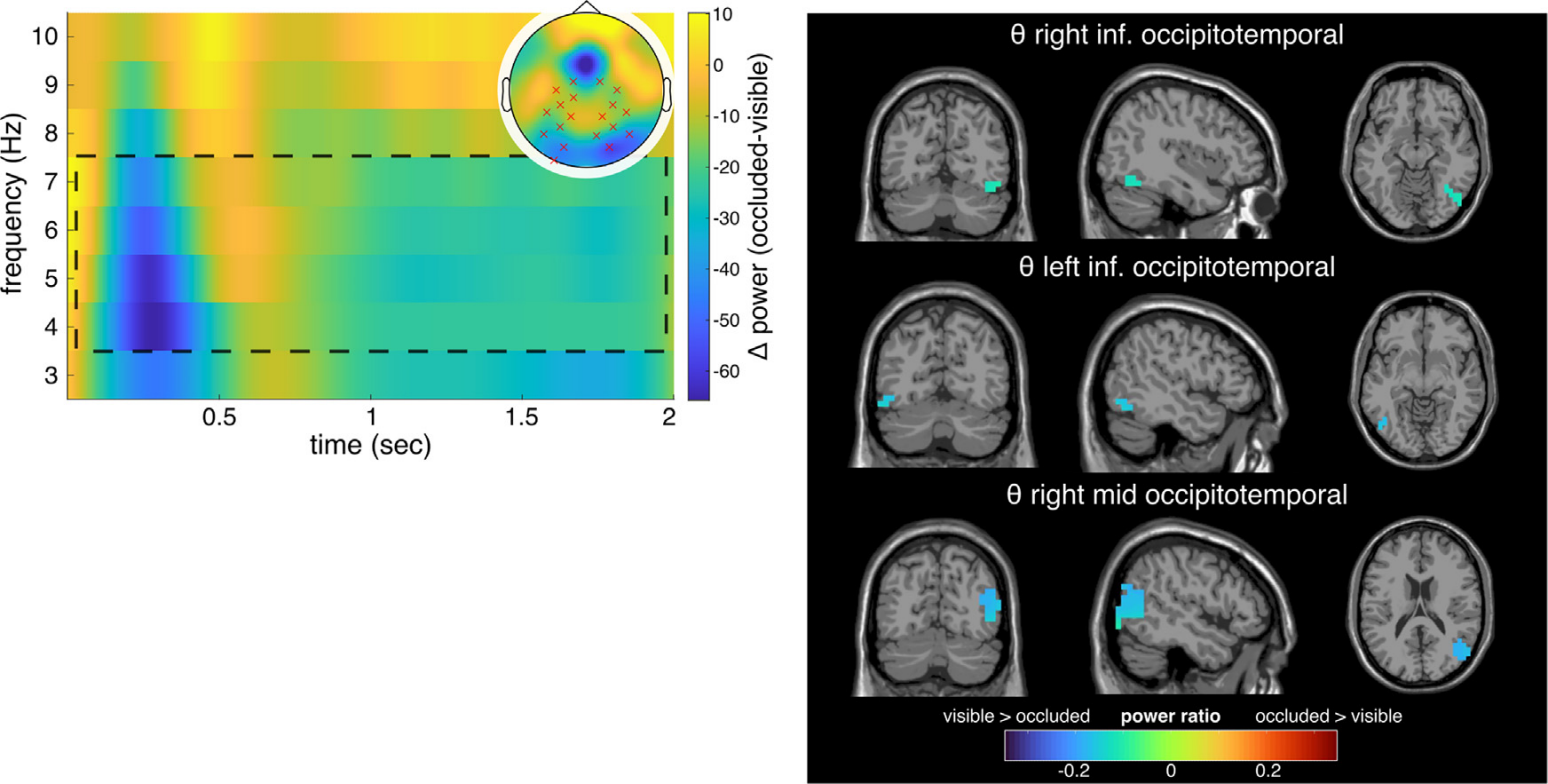
Results of the time-frequency and beamforming analysis for the contrast of occluded and visible cursor (occluded-visible) in the pursuit-occlusion paradigm Theta band power differences (occluded-visible) for significant electrodes in cluster-based permutation test on the left. The topography for the theta power difference is shown in the top right corner. Significant electrodes are highlighted with red crosses for negative and black crosses for positive differences. Significant negative power differences (visible > occluded) were found for the entire time interval (0–2 s). The dashed lines approximately enclose the significant time interval and frequency range. On the right, source activity differences for the theta frequency band are depicted in the clusters identified by the DBSCAN algorithm.

The contrast was calculated using the difference between the occluded and visible conditions (occluded-visible). Hence, a positive cluster indicates higher power in the occluded compared to the visible condition (Figure 10). Reconstructing the source-level activity in the theta band for this contrast, three neuroanatomical regions revealed activity modulations. Activity modulations were evident in the right-hemispheric inferior occipital and inferior temporal areas (BA19, BA37) and the inferior occipitotemporal cortex of the left hemisphere (BA19, BA37). Moreover, activity modulations were evident in the right hemisphere’s middle temporal and occipital cortex (BA19, BA21, BA37) (Figure 10). No significant clusters were identified for the alpha or beta band.

### Time domain analyses

As for task one, we analyzed whether there is a significant difference between ERPs following target direction changes. Therefore, we epoched the data 0 to 750 ms after target direction change. We contrasted trial segments during which the cursor was occluded with trial segments during which the cursor was visible. A cluster-based permutation test was performed over the whole epoch duration with a minimum cluster size of four electrodes.

**Figure 11:**
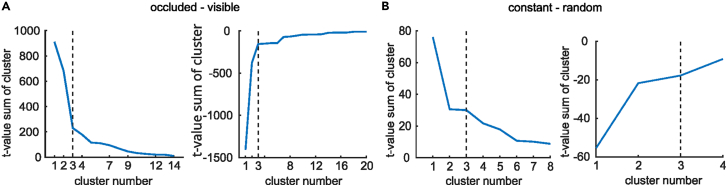
Scree plots of t-value sums per cluster Figure (A) shows the absolute t-value sums of each significant positive (left) and negative cluster (right), respectively, of the condition, occluded-visible. Figure (B) shows the same for the clusters of the contrast constant – random (positive left, negative right). The dashed line indicates our adjusted elbow criterion for cluster selection (see STAR Methods section for details).

The test yielded *N* = 20 significant negative clusters and *N* = 14 significant positive clusters. Positive clusters indicate that the ERP resulting from the occluded condition was significantly more negative than the ERP of the visible condition in the respective time window and for the respective electrodes. To decide which clusters are relevant to analyze in more detail, we inspected a scree plot of each cluster’s sum of t-values (see Figure 11). The cluster sum of t-values is the respective summed t-values of all the t-tests for each combination of spatial and temporal points that belong to a cluster. Using an adjusted elbow criterion, we decided to only further investigate the three largest clusters in each direction based on their respective t-value sums.

**Figure 12:**
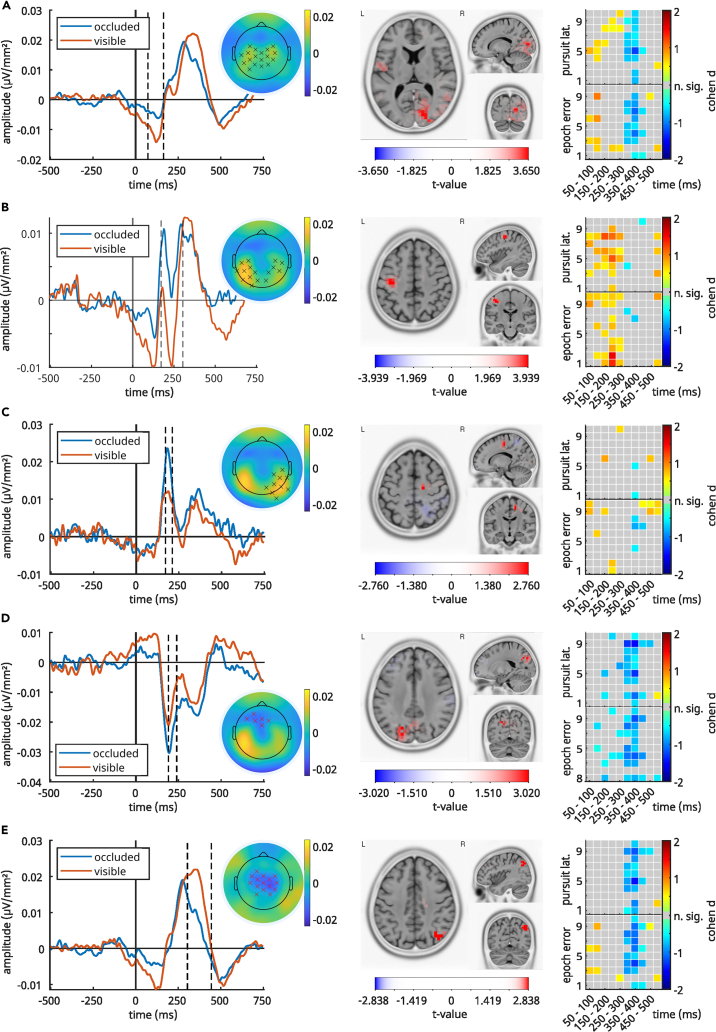
Relevant clusters in the occluded-visible contrast From Figure (A) to Figure (E), this figure visualizes the clusters detected by the cluster-based permutation test in the condition occluded-visible that are relevant for interpretation. In the left column are ERP plots contrasting the grand average ERPs after CSD transformation and weighting of all epochs. Time point 0 denotes the time point of a trajectory peak. The dashed line marks the time window of significant differences in the ERPs, as indicated by the cluster test. The topography next to the ERP plot marks the electrodes which were identified by the cluster test and whose signal is included in the ERP. The colors on the topography are the differences between the two conditions per electrode averaged across the whole time interval of the cluster. The central column shows the results of the sLORETA analysis of the time window of the cluster including all electrodes. The contrast between conditions occluded and visible is shown. Red voxels indicate that the activation in the occluded condition is significantly larger than in the visible condition; blue voxels indicate a significant effect in the opposite direction (note that the color scale range changes between Figures A–E). In the right column, the tracking performance vincentiles (x axis) are associated with the EEG data time bins (x axis). The colors of the cells indicate size and direction of the difference between conditions occluded-visible is for that vincentile-EEG bin combination, expressed in cohen’s d. Overall, the plots show no clear association between magnitude of condition differences and tracking performance. Cells with gray color denote t-tests between conditions that did not indicate significant differences.

**Figure 13:**
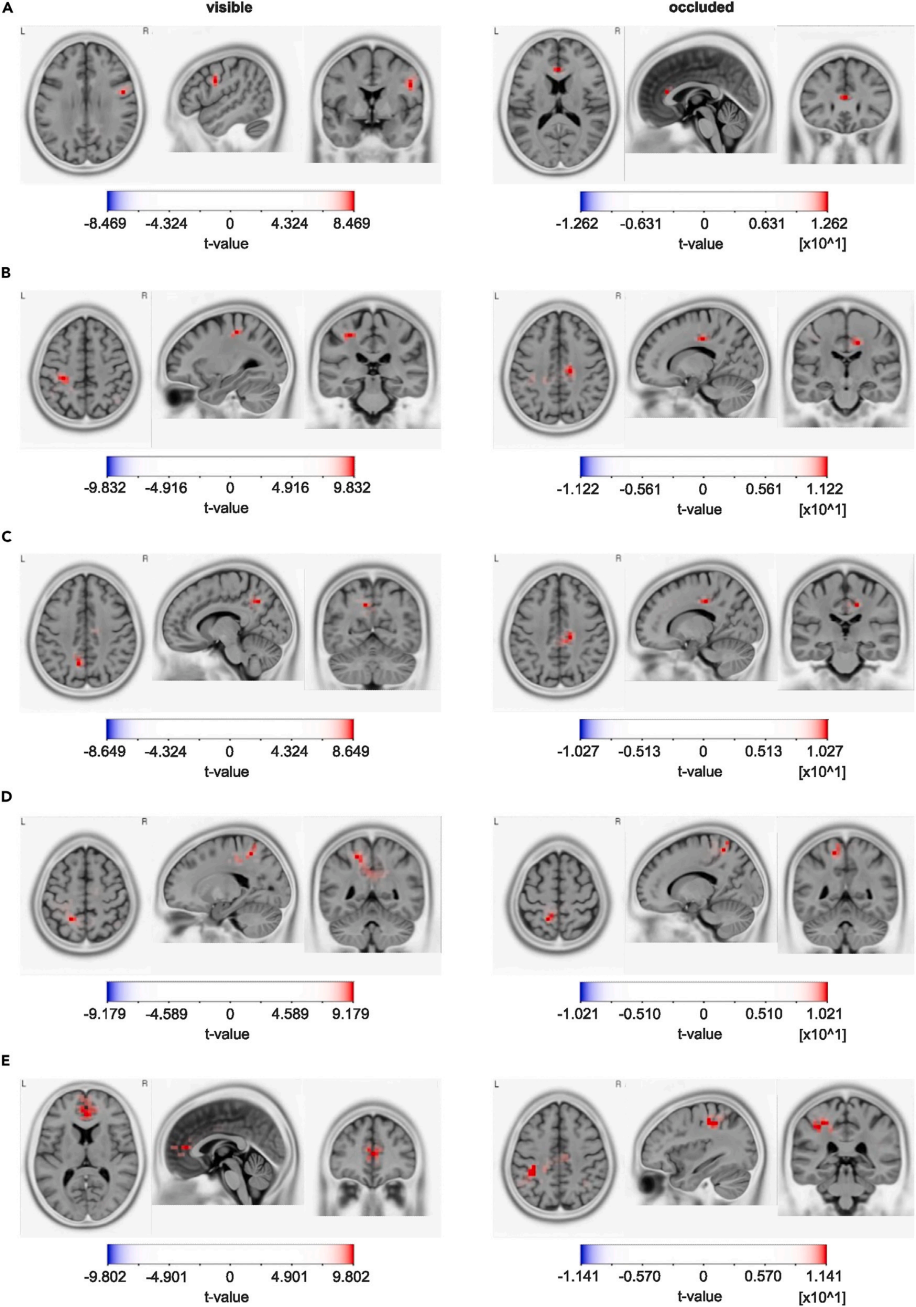
sLORETA estimates of Figure 12 clusters From Figure (A) to Figure (E), this figure visualizes significant sLORETA estimates of the conditions “occluded” and “visible” of the reported clusters A–E in Figure 12 against zero.

Figure 12 shows the ERPs, the result of applying an sLORETA algorithm to the occluded-visible contrast and the results of an analysis that reveals the association between tracking performance and EEG data (see section Single-trial time domain analysis and inter-relation with behavioral data). Figure 13 reveals the activity sources in occluded and visible conditions contrasted against zero.

As shown in Figure 12, the earliest positive clusters (Figure 12A) extended from 72 to 164 ms and over the central parietal electrodes. For the visible condition, sLORETA revealed significant activation in the precentral gyrus (BA6; MNI(x,y,z) = 50, 0, 30; p < 0.05) (Figure 13A left column). During occlusion, the most prominent activation was detected in the anterior cingulate (ACC, BA24; MNI(x,y,z) = 0, 30, 15; p < 0.05) (Figure 13A, right column). Contrasting visible-occluded in sLORETA revealed for this time range the most prominent activation in the cuneus (BA18; MNI(x,y,z) = 15, −85, 10; p < 0.05) (Figure 12A).

The second positive cluster (Figure 12B) was found at 172–212 ms at the right hemisphere’s parietal and occipital electrodes. Here, the sLORETA revealed that for the visible condition, activation differences in the precentral gyrus (BA4; MNI(x,y,z) = −30, −30, 50; p < 0.05) and during occlusion in the cingulate gyrus were evident (BA24; MNI(x,y,z) = 15, −20, 40; p < 0.05) (see Figure 13). Contrasting both conditions, the sLORETA revealed the most prominent activation in the precentral gyrus (BA4; MNI(x,y,z) = −40, −20, 50; *p* <. 05) (see Figure 12B).

The third positive cluster (Figure 12C) lasted from 172 to 304 ms and included bilateral parietal and central electrodes with sources in the precuneus (visible; BA7; MNI(x,y,z) = −10, −60, 40; p < 0.05) and the cingulate gyrus (occlusion; BA31; MNI(x,y,z) = 15, −25, 40; p < 0.05) (see Figure 13B). The contrast of both was in the medial frontal gyrus (BA6; MNI(x,y,z) = 15, −15, 55; n.s.) (Figure 12C).

The earliest negative cluster (Figure 12D) started at 192 ms and ended at 240 ms and extended over frontal medial electrodes with sources in the parietal lobe (visible; BA7; MNI(x,y,z) = −50, −60, 55; p < 0.05) and precuneus (occlusion; BA7; MNI(x,y,z) = −15, −50, 60; p < 0.05) (Figure 13). The contrast for this time range was located in the precuneus (BA7; MNI(x,y,z) = −20, −70, 35; p < 0.05) (Figure 12D).

Of note is that clusters C and D show very similar ERP waveforms, only in opposite orientations (D is negative while C is positive). Also, the locations of the electrodes that form these clusters are essentially mirrored across the midline of the head, indicating that the same neuronal source might be producing both activation patterns.

The final and second negative cluster (Figure 12E) was detected between 304 and 444 ms and included medial central, medial frontal, and medial parietal electrodes. Its sources were located in the anterior cingulate cortex (visible; BA32; MNI(x,y,z) = 0, 40, 10; p < 0.05) and inferior parietal lobule (occlusion; BA40; MNI(x,y,z) = −35, −35, 45; p < 0.05) (see Figure 13). sLORETA revealed the most prominent activation for the contrast of both conditions in the inferior parietal lobule (BA 39; MNI(x,y,z) = 40, –70, 40; n.s.) (Figure 12E). Cluster E encompasses almost the same electrodes as cluster A and therefore results in very similar ERP waveforms, revealing significant condition differences in two discrete time windows. Investigating cluster E more closely, there was a positive peak in the ERP in the occluded condition, which showed almost the same amplitude as the visible condition but at an earlier latency. In an exploratory analysis, a paired two-tailed t-test comparing the occluded and visible ERP peak latencies showed that the peak in the occluded condition occurred significantly earlier (mean = 290 ms) than the peak in the visible condition (*mean* = 345 ms; *t*(35,99) = −3.69, *d* = −1.17).

### Single-trial time domain analysis and inter-relation with behavioral data

As mentioned in the previous section, we examined whether the ERPs of the occluded and visible conditions in the significant clusters were related to the tracking performance. To this end, we used the ten pursuit-latency and epoch-error vincentiles for the occluded and visible conditions. The results are shown in Figure 12 (the rightmost plots in each line). We grouped the ERPs of the epochs associated with the respective vincentiles and averaged them across the electrodes of the clusters. We then split this average ERP into 50 ms time bins and averaged the ERP across the respective bins. The first bin started at 50 ms after a target direction change, and the last (10th bin) ended at 550 ms. This resulted in a 10x10 vincentile-bin matrix of combinations per cluster and error measure, each containing one average value per subject and condition. We then performed a t-test on each matrix cell and compared the occluded with the visible condition. Thus, we examined whether there were significant differences in the ERP related to the tracking task performance and established an association between tracking performance and neuronal processes. Figure 12 shows that results from the vincentile analysis and the cluster-based permutation ERP analyses only converge for clusters B and E. More precisely, only for these clusters, the vincentile analysis revealed effects in the same time windows that were also obtained for the cluster-based permutation tests. For cluster B, the cluster-based permutation tests revealed differences between the visible and the occluded condition between 172 and 212 ms, and significant differences in the vincentiles (see color shading in the rightmost plot in Figure 12B) were also evident in this time window. However, a scaling between the effect between the occluded and the visible condition (expressed using Cohen’s d) and the degree of behavioral tracking error (measures using epoch error or pursuit latency) was not evident. For cluster E (Figure 12E), the cluster-based permutation tests revealed differences between the visible and the occluded condition between 304 and 444 ms. Significant differences in the vincentiles were also evident during this time window. Since all other clusters did not reveal a consistent pattern of results between cluster-based permutation tests and vincentile analysis (also including the behavioral data), we consider the processes reflected in clusters B and E to be the most important.

## Discussion

The presented experiments provide detailed insights into the neurophysiological mechanisms underlying continuous action monitoring and fill a critical gap in knowledge of how the brain accomplishes action monitoring in ecologically more realistic situations that are not characterized by relatively short-lasting and regularly reinstating monitoring demands.^1^

### Tracking performance results

Sensorimotor demands on pursuit tracking increase when visual feedback on the cursor position is missing, which is indicated by the higher tracking error during cursor occlusion. However, participants reacted faster to target direction changes during cursor occlusion. This pattern of faster reaction times and lower tracking accuracy points to a potential speed-accuracy trade-off. Moreover, we found significant differences in the tracking behavior during the first 500 ms of the first random trajectory segment compared to the first 500 ms of the middle, i.e. constant trajectory segment. Adjustments of sensorimotor processes seemed to be particularly necessary during the first 500 ms of each trial. This finding led us to compare the first 500 ms of the random and constant trajectory separately from the rest of the trial.

### Time-frequency results

Due to the observed behavioral adjustments during the first 500 ms of each trial, the time-frequency data analysis during pursuit tracking was constrained by the different time intervals for the random and constant trajectory segments (see Figure 4). Within the first 500 ms, theta, alpha, and beta band activity significantly differed between the constant trajectory at the middle of the trial and random trajectories at trial onset, and power in the examined frequency bands was significantly larger while tracking random trajectory compared to constant trajectory. For all examined frequency bands, activity modulations were sustained over the entire 500 ms period, and the beamforming analysis revealed activity modulations in superior parietal areas and the precuneus (BA7). These brain regions are involved in processes contributing to the selection of motor responses.^48^^,^^49^^,^^50^^,^^51^ Superior and posterior parietal areas are known to integrate perception and action by binding sensory and motor information into a sensorimotor representation (program) necessary for goal-directed action.^18^ When faced with a random trajectory at trial start and when it is thus not possible to efficiently plan the specification of the sensorimotor program (incl. its execution) ahead of time, it is evident that activity in these regions has to be increased, compared to a situation where sensorimotor processes can be planned ahead of time (i.e., a constant trajectory during ongoing tracking). Crucially, three distinct frequency bands are involved in these mechanisms (i.e., theta, alpha, and beta band activity), each potentially playing specific roles in information processing as discussed in the following section. Out of these three frequency bands, the beta band activity is likely to play a unique role since beta band activity is not only associated with the superior parietal cortex and the precuneus (BA7) but also with superior frontal regions, including the supplementary motor area and the premotor cortex (BA4, BA6). Both the parietal regions and the observed modulations in superior frontal regions have been attributed to be central in the specification of complex sensorimotor programs and their monitoring.^25^^,^^26^ The obtained findings specify the associated oscillatory architecture of these processes and the functional relevance of these brain structures in continuous action monitoring.

As mentioned, beta band activity supports attention to upcoming motor tasks,^21^^,^^22^ premotor mechanisms guiding motor actions and likely the computation of the “status quo.”^24^ According to the status quo interpretation, the continuous maintenance of a sensorimotor program depends on predicting what is likely to happen soon to adapt the sensorimotor program. These processes must be intensified when faced with situations where the sensorimotor program needs almost permanent adjustments (i.e., in random trajectories). The observed modulations in theta and alpha band activity possibly support these beta band activity-related processes. This supporting role of alpha band activity is likely because alpha band activity likely reflects processes of gating and the controlled access of incoming information to a domain-general “knowledge system” (i.e., not restricted to perception, attention, working memory, and long-term memory) containing integrated information.^27^ Beta band activity may use the information gated through parietal alpha band activity to adapt the sensorimotor program during pursuit tracking. Crucially, this necessitates that incoming information is sampled and provided for the alpha band activity-controlled gating. This may be the role of the observed superior parietal theta band activity modulations. Aside from cognitive control and surprise signaling in medial/superior frontal cortices,^8^ posterior theta band activity plays a role in attentional control^16^ and especially the parietal source of theta band activity fits conceptions of non-spatial attentional processes enabled by superior parietal and precuneus regions.^18^ Moreover, several lines of evidence suggest that information sampling follows a theta rhythm.^52^^,^^53^ The theta band activity was stronger during the random trajectories at trial onset than constant trajectories in the ongoing trial. The finding substantiates the increased theta band activity-related information sampling during the initial period of pursuit tracking that in experiment 2, in which the visibility of the to-be-tracked trajectory was the central experimental manipulation, only theta band activity in the ventral stream was modulated in areas long-known to be central in the processing of visual motion information,^54^ as induced by visible tracking cursor movements.

At this point, it should be critically noted that the observed beta band activity difference occurs at the boundary between the alpha and beta band. However, we assume that the observed beta activity does not represent an extended alpha band activity, since we find different neuroanatomical sources for both activity differences and our experimental manipulation is likely to induce beta band activity differences.

Of note, after the initial 500 ms, only modulations in theta band activity were evident, suggesting that processes reflected by alpha and beta band activity in the initial period of pursuit tracking became less important. This can be attributed to sensorimotor learning and adaptation processes known to occur during pursuit tracking.^5^ This is not to say that processes reflected by alpha and beta band activity are no longer involved—they are just not differentially modulated between occasions imposing higher or lower demands on sensorimotor processes during pursuit tracking. Crucially, however, the quality of theta band activity modulation changed considerably compared to the initial period of pursuit tracking (i.e., the first 500 ms). Opposed to superior parietal theta band activity in the initial period of tracking, theta band activity modulations were stronger during constant than random trajectories and medial superior frontal structures (BA6) (instead of superior parietal ones) were associated with the modulations. The source in the medial superior frontal gyrus reflects a pattern observed in commonly used experiments to examine action monitoring,^8^^,^^9^ that is where relatively short-lasting and regularly reinstating monitoring demands are evident.^1^ Based on data from such approaches, it has been coined that theta band activity likely reflects a “surprise signal” necessary to adapt actions.^8^ For the pursuit of trajectories, tracking performance varies,^28^ depending on acquired implicit knowledge and the predictability of the target trajectory.^3^ Therefore, the finding that theta band activity is higher during constant than random trajectories can be explained well. Only when it is possible to build an expectancy of how events unfold, it can emit a surprise signal. Such expectancies cannot reliably be built when the trajectory is random.

### Time domain results

Especially after changes in the direction of the target trajectory, adaptive processes must be triggered to reduce tracking error. Avoiding the reduced temporal resolution of the time-frequency analyses, the ERP analysis provides insights into processes that happen on these occasions. The results were only reliable for task 2. Here, we consider the processes reflected in clusters B and E (Figures 12B and 12E) to be most important because only these clusters revealed a consistent pattern of results between cluster-based permutation tests and vincentile analysis (also including the behavioral data). For none of the clusters did the effects at the neurophysiological level scale with the degree of behavioral performance.

Cluster B (Figure 12B) resembles a P2/N2-like ERP complex that was larger for occluded and visible trials. Previous studies suggest that the P2 likely reflects resource allocation processes.^36^^,^^37^^,^^38^ Thus, resource allocation processes are intensified in the occluded condition and seem to take place in premotor cortices (BA4) as suggested by the sLORETA analysis. This is reasonable since, in the occluded condition, sensorimotor integration processes become complicated through the lack of visual information otherwise useful to inform motor program formation. Resource allocation processes may be predominantly needed to enable motor aspects of pursuit tracking in the absence of sensory information but are not decisive for the performance during tracking. This interpretation is corroborated by two other findings of this study: First, the analysis of tracking performance vincentiles and ERP data revealed that there was neither an inter-relation between the P2 amplitudes (locked at the trajectory peak) and the magnitude of the tracking error nor a relationship between the P2 amplitude and the latency of the pursuit peaks following the trajectory turning points. Second, there were no modulations of the P2 amplitude (and other ERP components) depending on whether there was a constant or a random trajectory to be tracked—in spite of modulations of behavioral tracking performance. Possibly, resource allocation processes are not volatile enough to be modulated during tracking and only provide a defined amount of processing capacity, which is then used by other processes that we find reflected in the theta, alpha, and beta band activity. Besides cluster B, effects were also evident in cluster E (see Figure 12E) between 304 and 444 ms, suggesting that the P3 ERP component is modulated as well. The P3 in response control paradigms likely reflects the mapping of the stimulus input onto the appropriate motor output.^40^^,^^55^ In line with that, P3 amplitudes are usually found to be smaller when demands on the mapping processes are increased^12^^,^^40^ and the herein found source of the difference in the inferior parietal lobule (BA39) substantiates that the P3 is modulated. The source localization analyses revealed that the cortical source of the P3 cluster E in the occluded trials was associated with activity modulations in the inferior parietal lobule (BA40). In the visible trials, the source was located in the ACC (BA32). The occlusion effect (i.e., the difference between occluded and non-occluded trials) was also reflected in theta band activity modulations during pursuit tracking (see discussion above). Previous findings show that parietal areas contribute to the selection of motor responses,^18^^,^^48^ and evidence that superior and posterior parietal areas serve to integrate perception and action by binding sensory and motor information into a sensorimotor representation (program) necessary for goal-directed action^18^ are also well in line with the current findings.

In conclusion, our study provides new insights into the neural processes underlying continuous action monitoring and the regular reinstatement of monitoring functions, which are critical for goal-directed behavior. The superior parietal and frontal cortices play a crucial role in this process, with beta band activity maintaining the sensorimotor program, and theta and alpha bands supporting attentional sampling and information gating, respectively. We also found that modulating sensorimotor demands through cursor visibility led to changes in the theta band and P2- and P3-like components. Our findings suggest that resource allocation mechanisms in prefrontal areas and stimulus-response mapping processes in the parietal cortex are crucial for adapting sensorimotor processes. This study fills a significant gap in our understanding of action monitoring by shedding light on the neural processes involved in continuous action monitoring, and thereby offers an alternative to the predominant reliance on experimental procedures imposing short-lasting and regularly reinstating monitoring demands.

### Limitations of the study

While the current study provides details and in-depth insights into the role of specific neural oscillatory activity, it does not reveal insights into the causal relevance of the oscillatory activity and the associated functional neuroanatomical structures of the processes focused in this study. This should be focus of future studies using non-invasive brain stimulation approaches.

## STAR★Methods

### Key resources table

**Table:** 

REAGENT or RESOURCE	SOURCE	IDENTIFIER
**Software and algorithms**		

MATLAB 2021b	https://de.mathworks.com/products/matlab. html	RRID:SCR_001622
BrainVision Recorder	https://www.brainproducts.com/productdetails.php?id=21	RRID:SCR_016331
PsychoPy	http://www.psychopy.org	RRID:SCR_006571
EEGLAB	http://sccn.ucsd.edu/eeglab/index.html	RRID:SCR_007292
Fieldtrip	https://www.fieldtriptoolbox.org	RRID:SCR_004849

**Deposited data**		

Raw data behavior	This paper	https://osf.io/pv49m/
Raw data EEG	This paper	https://osf.io/pv49m/

### Resource availability

#### Lead contact

Further information and request for resources should be directed and will be fulfilled by the lead contact, Christian Beste (Christian.Beste@uniklinikum-dresden.de).

#### Materials availability

There are no newly generated materials.

### Experimental model and subject details

#### Participants

The initial study sample consisted of N = 34 healthy adults. Three participants were excluded due to incomplete data recording. One participant was excluded since the behavioral data indicated that this participant was not following the instructions, resulting in a final sample of N = 30 subjects aged from 20 to 30 years (on average 25.42 ± 3.04 years). This sample comprised N = 11 male and N = 19 female participants who were right-handed. All participants had normal or corrected-to-normal vision and reported no neurological or psychiatric illness history in the past six months. The study was conducted in accordance with the Declaration of Helsinki and approved by the IRB of the TU Dresden (EK 390082019). The participants were compensated for their participation and provided written informed consent.

### Method details

#### Task

In this study, the participants executed two pursuit-tracking tasks, with the order of the tasks randomized across participants. In both tasks, participants were asked to track a moving red square (the target) using an equally sized crosshair (the cursor) controlled by a joystick (Thrustmaster T16000M) during each trial. The objective was to maintain the cursor on the target as accurately as possible (see Table S1 for precise instructions). Participants could only control the vertical movement of the cursor, with the joystick’s deflection proportionally affecting the cursor’s position on the screen: maximal joystick deflection moved the cursor to the edge of the screen, while no deflection centered the cursor. The target, a 32 x 32-pixel red square (0.79° visual angle), moved across the screen along a three-segment trajectory, while the cursor and target maintained the same horizontal position. Each segment was created by concatenating three sine and cosine terms^28^:

$$f\left(x\right)=\sum_{i=1}^{3}a_{i}\cdot \sin\left(i\cdot x\right)+b_{i}\cdot \cos\left(i\cdot x\right)$$

The first and last segments had randomly generated coefficients between −40 and 40 pixels which ensured that the trajectory did not reach the top or bottom of the screen. The central segment remained constant across all trials, with fixed coefficients: a1 = 37, a2 = −3, a3 = 26, b1 = 23, b2 = −15, and b3 = −9. Participants were not informed of the constant central segment. Smooth transitions between the segments were achieved by placing a 30-pixel gap between them and using cubic splines for interpolation. The target began at the center of the screen and moved either left or right at one of three possible velocities, which remained constant within a trial. The direction and velocities of the target’s movement were counterbalanced, with each combination repeated twelve times, resulting in a total of 72 trials. Each trial was separated by a white fixation square of the same size as the target, which appeared for a random duration averaging 1.5 s. After every ten trials, participants received a 30-s break. The average trial lasted 12 s, making the total task duration approximately 20 min.

In task 1, participants completed the basic pursuit-tracking paradigm as described above. In task 2, the paradigm was modified by occluding the cursor for 2 s every 4 s during each trial, starting with a visible cursor. This alteration aimed to investigate the effect of intermittent cursor visibility on participants' tracking performance. The task was programmed using PsychoPy2.

#### EEG recording and preprocessing

For the EEG recording, we used EasyCaps with 60 passive Ag-AgCl electrodes and BrainAmp amplifiers from Brain Products Inc. (Brain Products GmbH, Gilching, Germany). The electrode placement was an equidistant setup based on the 10%-system with a reference electrode at position Fpz.

The EEG recording was carried out with a sampling rate of 500 Hz. Later, offline down-sampling to 250 Hz was applied. The impedances of all electrodes did not exceed 10 kΩ. EEG data were pre-processed using EEGlab^56^ on MATLAB 2021b (IBM Mathworks Corp.). Firstly, the data were high-pass filtered at 1 Hz using a Hamming windowed FIR filter. A line-noise filter at 50 Hz was applied using the *pop_cleanline* plugin for EEGlab. Then, flat channels and those with minimum channel correlation were removed. A low-pass filter (Hamming windowed FIR filter) for 40 Hz was applied. Subsequently, the removed channels were interpolated using a spherical method. This was done to ensure all participants have the same number of channels to facilitate further analyses. Next, the data were re-referenced to the common average. Before conducting the ICA, the appended data was detrended, and artifacts in this dataset were removed using a joint probability method (as implemented in *pop_jointprob*.^56^ An independent component analysis (using an extended infomax algorithm) was conducted for the appended trials of both the pursuit-tracking and the pursuit-occlusion task. To identify all components not labeled brain activity, we applied the “IClabel algorithm.” All components not labeled brain activity with at least 50% probability were removed automatically. To further check for artifactual components, we inspected the topography of the first five remaining components visually. Detected artifactual components were excluded and all remaining components were applied to the data of each task respectively. Subsequently, the EEG data were parallelized with the behavioral data of each paradigm, thereby excluding all EEG data for trials that were excluded based on the behavioral data.

### Quantification and statistical analysis

#### Calculation of tracking performance

Since the cursor and target coordinates were recorded at 60 Hz and the EEG data was processed with a sampling rate of 250 Hz, we up-sampled the coordinates to 250 Hz using cubic spline interpolation. Then, two error measures describing behavioral performance were calculated: tracking error and pursuit latency. The tracking error quantifies how accurately participants were tracking throughout a trial/epoch. It is defined as the root mean squared error (RMSE) between the target y axis position and the cursor y axis position for each sample. The pursuit latency is the time that passes between a target direction change and the following direction change of the cursor. To obtain this, peaks in the target trajectory and pursuit tracking coordinates were identified first. For the target trajectory, all peaks that exceeded a prominence (how much the peak’s height stands out compared to surrounding peaks) of 1% of the screen size were defined as peaks that the participants likely perceived and could respond to. For the pursuit, which naturally contains more jitter due to motor noise, a prominence threshold of 5% of the screen size was defined as the threshold above which a change in joystick direction implies an intentional direction change. To calculate the pursuit latency, we matched the trajectory peaks with the pursuit peaks that followed them. We considered a pursuit peak valid if it occurred between 80 ms and 300 ms after a trajectory peak (since the trajectory peaks are, on average, 300 ms apart) and was in the same direction as the trajectory peak (both local minima resp. maxima). The time between the trajectory peak and a valid pursuit peak reflects the pursuit latency. Of 50,488 epochs, 29,593 have a valid pursuit latency (58,61%). Subsequently, we used the error measures to exclude trials during which participants did not follow the experiment instructions adequately: the tracking performance in all the trials with a maximum tracking error more than three standard deviations above the mean maximum tracking error per trial was inspected visually. The trial was excluded if the pursuit did not follow the target trajectory. Finally, upon inspection of the distribution of the frequency of tracking errors over the trial, we noticed that the distribution peaked more during the first 500 ms as compared to the rest of the trial (see Figure 2). Subsequently, we quantified this difference in the randomness of the error distribution by calculating sample entropy^44^ using the MATLAB function. Sample entropy quantifies how complex a time series is. It is the negative natural logarithm of the probability that a pattern of samples of length m (standard: m = 2) found later in the time series will also be the same for the next element. For two samples to be regarded as “the same,” they need to be less than the similarity criterion “r” apart, which is traditionally set to 0.2 times the standard deviation. Notably, sample entropy does not depend on the length of the investigated time series. For this analysis, we z-transformed each segment to be investigated for each trial individually. This made the trial segments by different participants comparable and also allowed us to know the standard deviation, which allowed us to use the same similarity criterion across all trials, avoiding introducing bias in the analysis based on a variable similarity criterion. Subsequently, we compared the sample entropy values of the first 500 ms to the sample entropy values of the following 1500 ms. Since the sample entropy values of the first 500 ms are not normally distributed (see Figure 2), we used a Wilcoxon rank-sum test to compare the two conditions. The significant difference between the conditions can be easily explained by investigating images of single trial pursuits plotted against trajectory courses. The tracking error in this initial interval, in contrast to the interval after the initial 500 ms, mainly depends on the target’s movements. It is either increasing (by moving away from the cursor) or decreasing (by crossing the cursor) the tracking error. We analyzed the respective EEG data from the first 500 ms separately since this time interval likely reflects sensorimotor calibration processes needed to engage in continuous action monitoring required during pursuit-tracking.

#### Behavioral data significance tests

To test whether there are differences in tracking performance between conditions, we ordered the behavioral data of each epoch into ten vincentiles per subject and condition, which means that we first ordered the error measures (epoch error and pursuit latency) by size and then split the ordered data into ten equal-populated bins. The vincentile procedure^57^^,^^58^ is preferred above analyzing quantiles because it assures the independence of sample size within the analysis bins. We then averaged the data inside each bin so that every subject was represented by one value per condition vincentile. For each vincentile, we performed a paired two-tailed t-test with an alpha level of 0.001 by taking 5,000 bootstrap samples from the subject sample data, comparing the conditions occluded and visible as well as constant and random. Finally, we performed an FDR correction with *q* = 0.05 on the resulting ten p values per comparison to reduce the risk of false positives.

#### Time-frequency decomposition

For the time-frequency analysis, a baseline correction was conducted for all trials, using the interval before the trial start (−750 ms to trial start), i.e., the time interval in which only the fixation cross was presented, as a baseline. Then, the data were epoched considering the entire trial length (i.e., 13 s from trial start trigger) and bad trials were rejected using a joint probability method (as implemented in *pop_jointprob*.^56^ The data were then segmented according to critical experimental manipulations: for task 1, we divided the data into random and constant trajectory segments, each 3 s long. For task 2, we segmented the data into occluded and non-occluded trajectory parts, each 2 s long. After conducting the preprocessing in EEGlab, the data was imported to the Fieldtrip Toolbox.^59^ A time-frequency analysis was performed using Morlet wavelets with a width of 5 Gaussians for the theta (4–7 Hz), alpha (8–12 Hz) and beta (13–30 Hz) frequency band.

Since the behavioral tracking data indicated different tracking behavior in the first 500 ms of each trial compared to the proceeding trial (see Results section), we separated the trajectory segments for the trial start and the proceeding trial, respectively. Since every trial starts with the random trajectory segment, we separated the random segment into a trial start interval (0–500 ms, 'first interval') and an interval for the proceeding random trajectory (500–3000 ms, 'second interval'). To achieve comparable trial segments of equal length for random and constant trajectory, we also segmented the constant trajectory interval in two parts, with the first interval from 0 to 500 ms of the constant trajectory and the second interval from 0 to 2500 ms of the constant trajectory. Here, the start of the segment was also included for the second interval since the behavioral difference was only observed at the trial start and the constant trajectory segment always appeared in the middle of a trial. For a detailed overview of the segmentation, see Figures 3 and 7).

To investigate significant differences in the power of the three frequency bands, we conducted cluster-based permutation tests with 1000 Monte-Carlo iterations and a cluster alpha level of 0.001. For task 1, we compared the constant and random trajectory segments for the first and second time interval (i.e. first 500 ms and 500 ms onwards) respectively. For task 2, we compared the occluded and non-occluded segments.

#### Beamforming analysis

Using dynamic imaging of coherent sources (DICS) beamformer,^45^ we reconstructed source-level activity for the frequency bands and contrasts that showed significant power differences in the cluster-based permutation tests. For that, we extracted the cross-spectral density matrix for each condition using a Fast Fourier Transformation (FFT) on the alpha (8–12 Hz), beta (13–30 Hz) and theta (4–8 Hz) frequency bands. Based on the appended data for each contrast, we calculated a common spatial filter with the DICS beamformer using a 5 mm grid based on the template-based forward model implemented in the Fieldtrip toolbox. The common spatial filter was applied to the respective conditions in a second step. The contrast was calculated as the source power difference divided by the sum of both conditions, thereby normalizing the difference using the following ratio:

$$r=\frac{{power}_{condition1}-{power}_{condition2}}{{power}_{condition1}+{power}_{condition2}}$$

Based on the assumption that there is an equal noise distribution in both conditions, this ratio mitigates potential bias due to noise and diminishes the effect of outliers.^60^ We then used the “Density-Based Spatial Clustering of Applications with Noise” (DBSCAN) algorithm^61^ to identify clusters of alpha-, beta- and theta-band for the significant contrasts, using the implementation in MATLAB. The clusters were identified with the respective ratio of both conditions for each contrast. This approach limits the source activity to demarcated functional neuroanatomical regions.^17^^,^^46^ Depending on the direction of the difference, the most positive or negative 1% of the power distribution of all voxels within labeled neuroanatomical regions on the Automatic Anatomical Labeling (AAL) atlas was used for the DBSCAN. As the epsilon parameter, denoting the neighborhood search radius, we used 1.5 times the edge length of each voxel. The clusters identified by the algorithm were inspected visually and defined based on the number of voxels and anatomical labels.

#### Time-domain analysis

For the time domain analysis, the EEG data were epoched around the trajectory peaks, with epochs extending from −500 ms to 750 ms. A baseline correction was applied to the first 500 ms of each epoch. As with the time-frequency data, bad trials were rejected using a combination of the joint probability method and the same criteria used for the time-frequency analysis. The average tracking error and the pursuit latency were calculated for each epoch. To improve the topographical localization of scalp surface potentials, we used a spherical spline algorithm to compute the current source density (CSD), as implemented in the CSD Toolbox.^62^ We used the default parameters of this toolbox. Epoching was done for each experimental version of the tracking task separately. For task 1, epochs were created with time point zero denoting the trajectory peak in the first random segment of each trial or the constant segment in each trial. For task 2, epochs around peaks in the trajectory were created and sorted depending on whether the cursor was occluded or non-occluded at the time of the peak. The data were weighted depending on the number of epochs per participant to account for an unequal number of epochs between participants. The weight per subject was determined by calculating the median of the number of trials per subject for each condition and dividing it by the number of trials that subject had. This weight was then multiplied with the EEG data of the respective condition and subject. The condition-specific grand average of all epochs was calculated. Cluster-based permutation tests across the epoch interval from 0 to 750 ms with 3,000 Monte Carlo iterations were performed to identify significant differences between conditions. A conservative alpha and cluster alpha level of 0.001, respectively, was chosen. Also, all clusters that found significant activation in less than four neighboring electrodes were ignored. The algorithm detected many clusters, which we further narrowed down by applying a multi-step selection process. First, we investigated a scree plot of the cluster t-value sums for both contrasts (constant - random and occluded - visible) and directions (positive and negative) (see Figure 11). The cluster t-value sums are the summed-up t-values of all t-tests that are organized in a cluster, so one test per electrode-sample combination. It is a measure of cluster magnitude in the dimension’s spatial extent, temporal duration and effect size. Since the “elbow” of the scree plot of cluster t-value sums was at the second or the third cluster in all scree plots, to establish consistency across contrasts, we chose to analyze the three largest clusters per condition further. Of these clusters, we investigated topo plots of the averaged ERP across the cluster time intervals alongside ERP plots of the averaged signal of the electrodes on one cluster. This led us to exclude all constant-random clusters from further analyses and one of the occluded-visible clusters for the following reasons, respectively. For one, we excluded all clusters that showed significant condition differences across a very short time (less than five samples), likely not reflecting meaningful physiological activity. Second, we rejected clusters that appeared more than 300 ms after the target direction change. The reason is that on average, after 300 ms, the following target direction change already took place, which means that activity after 300 ms is to more than 50% a mixture of activity related to the target direction change at time point 0 as well as activity related to the following target direction change.

#### Association of tracking performance and ERPs

We further analyzed whether the ERP time course after a target direction change was associated with the behavioral tracking performance. To this end, we used the behavioral tracking performance vincentiles and grouped the EEG data in 10 time bins, starting from 50 ms after a target direction change until 550 ms. For the EEG bins, we only used data from electrodes in significant clusters and from epochs with a valid pursuit latency. We chose a duration of 50 ms per EEG data time bin as a reasonable compromise between signal to noise ratio and time resolution, with 12–13 samples contributing to the average of a bin. We ended up with a matrix of EEG data for each subject and condition where each row represented a vincentile and each column a 50 ms time bin. Which results in a total of 100 vincentile-time cells. We calculated the average ERP amplitude for each vincentile-time cell. Thus, we obtained a representation of the association between the ERP time course following a target direction change and the different degrees of tracking performance. Akin to the test of the behavioral data, we subsequently performed a paired two-tailed t-test with 5000 bootstrap samples for each vincentile-time cell, which resulted in 100 tests per condition using an alpha level of 0.001. To reduce the risk of false positive findings, we performed an FDR correction with a q-value of 0.05. To quantify the degree of difference between the vincentile-time bins, we calculated cohen’s d for each cell.

#### Time-domain source localization analysis

The source localization of the time-domain data was carried out using the sLORETA software (standardized low-resolution tomography (sLORETA).^63^ sLORETA has been validated in numerous studies using brain stimulation-EEG and fMRI/EEG or structural imaging and yields reliable source estimations.^64^^,^^65^ sLORETA, as used herein, uses the single-subject averaged data (i.e., averaged across single trials in a specific condition) as input. The method is based on a realistic head model^66^ and partitions the intracerebral volume into 6239 voxels with 5 mm spatial resolution. Based on the MNI152 template, the standardized current density at each voxel is calculated to localize the sources of scalp electrical activities. In the current study, sLORETA was used to compare conditions in significant cluster time windows revealed by the cluster-based permutation tests in the time-domain analysis to locate brain regions activated between the contrasted conditions. For all comparisons, sLORETA’s built-in randomization tests, based on nonparametric statistical mapping (SnPM), were utilized to correct for multiple comparisons. For this, 5000 permutations were used. The significance level was set to 0.05.

## Data Availability

De-identified raw data for the behavioral analysis as well as raw EEG data have been deposited at Open Science Forum: https://osf.io/pv49m/. EEG data at different processing steps are available on reasonable request.
