# Supplementary material for: The neurophysiology of continuous action monitoring
Source: iScience. 2023 May 22;26(7):106939. doi: 10.1016/j.isci.2023.106939 (PMC10275727; doi:10.1016/j.isci.2023.106939)
Supplement: Document S1. Table S1 [file mmc1.pdf]

iScience, Volume *26*

Supplemental information

The neurophysiology of continuous action monitoring

Saskia Wilken, Adriana Böttcher, Nico Adelhöfer, Markus Raab, Sven Hoffmann, and Christian Beste

Supplemental Information

Table S1. On-Screen Task Instruction Sequence, related to Methods

Experiment	Instruction (actual)	Instruction (translation)
both	Im folgenden Experiment werden Sie ein kleines Quadrat sehen, welches sich nach einer kurzen Zeit entweder nach LINKS oder RECHTS bewegt.	In the following experiment, you will see a small square that after a short time moves either LEFT or RIGHT.
both	Zusätzlich erscheint eine Art Fadenkreuz, welches sich wie das Quadrat nach links oder rechts bewegt. Dies können Sie mit dem Joystick auf (nach vorne drücken) und ab (nach hinten ziehen) bewegen.	In addition, a kind of crosshair appears, which moves to the left or right like the square. You can move this with the joystick up (push forward) and down (pull back).
both	Ihre Aufgabe besteht darin das Fadenkreuz möglichst genau auf dem Quadrat zu halten. Versuchen Sie dabei so genau wie möglich zu sein.	Your task is to keep the crosshairs as accurate as possible on the square. Try to be as accurate as possible.
only 2	In regelmäßigen Abständen wird das Fadenkreuz für kurze Zeit unsichtbar.<br>Hier ist es Ihre Aufgabe, sich vorzustellen, wo das Fadenkreuz sich befindet, und weiter mit dem Joystick das rote Quadrat zu verfolgen.	At regular intervals, the crosshairs become invisible for a short time.<br>Here your task is to imagine where the crosshair is and continue to follow the red square with the joystick.
both	Bitte halten Sie den Joystick mit der rechten Hand.<br>Sitzen Sie bequem und versuchen Sie, nicht zu verkrampfen. Nutzen Sie die Pausen während des Experiments, um sich zu lockern.<br>Wenn Sie noch Fragen haben, wenden Sie sich jetzt an den Versuchsleiter.	Please hold the joystick with your right hand.<br>Sit comfortably and try not to tense up. Use the breaks during the experiment to loosen up.<br>If you have any questions, please contact the experimenter now.
both	Sie können die Aufgabe jetzt mit 6 Durchgängen üben.	You can now practise the task with 6 trials.
